# Experiential Lens in Nursing Education and Thriving Lāhui (Community): A Native Hawaiian and Pacific Islander Student Experience

**DOI:** 10.31372/20200503.1104

**Published:** 2020

**Authors:** Nainoa T. Gaspar-Takahashi, Edna R. Magpantay-Monroe

**Affiliations:** Chaminade University of Honolulu, Hawai‘i, United States

**Keywords:** experiential learning, service learning, problem based learning, Native Hawaiian, infographics

## Introduction and Background

The purpose of this descriptive paper is to explore examples of high impact practices in nursing education that affects the community of interest and more specifically from the perspective of a native Hawaiian and Pacific Islander student. High impact learning involving communities benefits nursing students by honing their critical thinking skills and compassionate way of knowing (Hill, 2017).

The native Hawaiian and Pacific Island lāhui (community) is the focus for the experiential learning experience conducted by the author, a 22-year-old sophomore nursing student, of mixed race (native Hawaiian, Asian, and Pacific Islander). He participated in a service-learning project and problem-based learning using infographics in his freshman year at a Catholic Marianist university. The student’s aspiration of contributing to his community is exemplified by his participation in service learning and health promotion activities that affects his lāhui (community). According to Shannon (2016), students are more likely to promote health in a community-based population that is closely connected to them. In the native Hawaiian community, “the family or kinship group (‘ohana) is central to native Hawaiian culture and is the method by which cultural values, history, rituals, and traditions are communicated” (Cabrera & Smith, 2018, p. 1). This paper describes how one student’s experiences are interconnected to the concept of ‘ohana (family). Scheiding and Cellini (2019) suggests that experiential learning can be valuable to native Hawaiian students as a form of active engagement especially when their indigenous culture is not incorporated into the course.

## Significance

The value of high impact practices such as service-learning and problem-based learning through use of infographics that supports experiential learning has many outcomes conducive to the production of strong well-rounded students. These outcomes include but are not limited to enhancement of personal and professional skills, increased self awareness and confidence, improvement in academic, and leadership skills (Carlisle, Gourd, Rajkhan, & Nitta, 2017). These outcomes can be beneficial for any nursing student but particularly to native Hawaiian and Pacific Islander students. According to Cabrera and Smith (2018), native Hawaiian students value the sense of well-being and have a holistic perspective and approach to their learning and practice.

At a Catholic Marianist university, service learning is at the core of the student educational experience. It is “a high-impact practice by which students learn through active participation in thoughtfully organized service which meets the needs of the community … It includes structured means for reflection on the service experience and helps to foster civic and corporate responsibility. As a pedagogy, service-learning emerges from experiential learning theory and encourages active student involvement in the learning process” (Chaminade University of Honolulu, 2020, para. 1). The value of service learning among native Hawaiian nursing students may originate from understanding their cultural beliefs, values, and attitudes (Caspersz & Olaru, 2017). Service learning translates to a strengthening of civic engagement and responsibility which are all important in native Hawaiian culture (Cabrera & Smith, 2018).

Infographics was a tool used for the problem-based learning (PBL) project. PBL projects create a tone of creativity by allowing students to solve a problem or issue through their personal lens. Infographics can explain a complex concept and are useful teaching and learning strategies for all levels of nursing students (Chicca & Chunta, 2020). The information presented in infographics can be useful in presenting key concepts to different types of learners. Students are able to use infographics as a learning-tool while providing educational information for peers and those in the community. Infographics is a great way to interact with others by including and addressing community needs that will promote wellness and best practices. The infographics or any project in a health-related field created by the native Hawaiian students usually promotes harmony and well being as part of their cultural heritage and values (Cabrera & Smith, 2018).

## Description of the Experiential Learning Projects

The first experiential learning project is a service-learning project which was completed at Mālama I Nā Ahupua‘a (MINA) where learning about the native Hawaiian culture was exemplified through the common core activities and beyond like the upland (heiau), a midland (lo‘i), and lowland (fishpond) activities (Mālama I Nā Ahupua‘a, 2020). The goal of the project is to allow students to develop a sense and responsibility of place (MINA). Native Hawaiian practices of sustainability are introduced through practical applications. Students completed at least 10 hours of service at different locations around the island of O‘ahu: Hālawa Valley and Kāko‘o ‘Ōiwi. At both locations, students contributed to the removal of invasive species of plants that poses a threat to native Hawaiian flora and fauna. At Kāko‘o ‘Ōiwi, students learned about the lo‘i (midland) patch water systems and the native kalo (taro) that grow there, and at Hālawa students learned about a special birthing stone in which mothers and midwives would birth the future of Hawai‘i’s children (MINA).

The second experiential learning is a PBL project using infographics which was completed during the first nursing course: Introduction to Nursing. The activity involves creating an individual student infographics that can be compared with another student’s infographics with focus on specific nursing topics/issues such as health promotion. The project culminated in creating a cohesive comparison of infographics which included native Hawaiian values, Marianist values, Quality Safety Education for Nurses (QSEN) competencies, Bachelor of Science Nursing (BSN) essentials, Institute of Medicine (IOM) competencies, and nursing theoretical frameworks. Students were able to intentionally choose their topics and some related their chosen topics to their service learning experiences. The project was completed at a Catholic University where values of native Hawaiian and Marianist are important. For the infographics project, the student author created infographics about health promotion. Health promotion is a primary prevention tool that is helpful in working with a diverse patient population (Kaholokula et al., 2018). The connection between the health promotion topic and native Hawaiian values are *hana ka lima* (work diligently together) and *lokahi* (harmony and unity). The teamwork and collaboration expressed in the completion of the project exemplifies native Hawaiian values of *kuleana* (shared responsibility).

## Discussion

Both experiential projects provided an intentional reflection that is essential as a future nurse. Service learning allowed students to appreciate their support system: the lāhui (community). There is a sense of gratitude to be able to give back to the community that students grew up in. The ability to inspire others, leaving their comfort zone to learn about other communities and recognize the strength of it can form deeper understanding and acknowledge the place students call home. If given an opportunity to present their experiential learning to their peers and communities, students will create a lasting impact on their chosen profession such as nursing. The nursing student author and the audience were impacted by the presentation through assimilation of new knowledge and consideration of the experience towards the community. The impact to nursing as a profession is the integration of evidence-based care to clinical practice.

The projects led to teamwork and collaboration by students and professionals from multidisciplines and multiethnic backgrounds. The collaboration among the team members fostered a sense of ‘ohana (family). This sense of ‘ohana (family) is the fabric of native Hawaiian culture which is important to the student author. The experience allowed for the ability to advance foundational knowledge as with many hands (*working as a community*), greatness can be achieved. The infographics experience, which focused on health promotion, allowed the student to make the connection to the service-learning project site. The use of infographics which helps simplify complex information to manageable chunks of information supported critical thinking skills and mindful way of looking at concepts important for nursing students. Despite many values of the projects to the student experiential learning, time was a barrier in spending additional valuable time with the community. Time was also a factor in collating reliable information for the infographics due to other school and life obligations.

## Implications

These two projects provide a strong foundation in the nursing educational journey of the nursing student through a mindful practice. Experiential learning and mindful approach allow for intentional learning that is meaningful to making informed decisions as a future nurse. Consideration for future research studies and projects can include the following: (1) a broader native Hawaiian nursing students perspectives on the impact of experiential learning to their nursing education, (2) the use of evidence-based tools and technology in teaching and learning of native Hawaiian and Pacific Islanders nursing students in enhancing their critical thinking and clinical reasoning skills that will help prepare them for professional practice and (3) the value of projects that enhances community engagement and lessons learned in alignment with the native Hawaiian values. Nursing student engagement in enriching projects is driven by their desire and motivation to apply their learning to their community of interest which then allows for better reflection and care for their lāhui (community).
